# SOP myasthenic crisis

**DOI:** 10.1186/s42466-019-0023-3

**Published:** 2019-07-29

**Authors:** Henning Stetefeld, Michael Schroeter

**Affiliations:** Department of Neurology, University of Cologne, Kerpener Str. 62, 50937 Cologne, Germany

## Abstract

**Introduction:**

The overall prevalence of myasthenic crisis is quite low at 30/1 million inhabitants because myasthenia gravis is a rare disease per se. But it should be noted that 15–20% of patients with myasthenia gravis experience at least one crisis in their lives. Most often, the crisis occurs within the first 2 years of the disease or is even the first manifestation of a yet undiagnosed myasthenia gravis in up to 20%.

Median duration of MC is about 2 weeks (median 12–14 days of ventilation) under sufficient treatment, but prolonged courses are not uncommon and often due to comorbidities and complications, so that about 20% are still mechanically ventilated after 1 month.

The lifetime risk of recurrence of a crisis is approx. 30%. Data on mortality differ between about 2–5% to even more than 16%. Lethal outcomes are almost never caused by the crisis itself, but because comorbidities or complications eventually become limiting.

**Definition:**

Myasthenic crisis (MC) is the life-threatening maximal manifestation of myasthenia gravis (MG) necessitating mechanical ventilation, supportive feeding and (neuro-)intensive care. Weakness may develop within minutes to days and encompass flaccid tetraparesis with immobility, severe dyspnea, respiratory insufficiency and aspiration. Globus events may be life threatening due to rapidly exhausting coughing and swallowing.

**First steps: immediate measures:**

● Check and secure vital functions

**Comments:**

● not applicable

**Conclusion:**

The main symptom of (imminent) myasthenic crisis is the rapidly progressive weakness of the respiratory and bulbar muscles, which lead to a decompensation with aspiration and respiratory insufficiency. Clinical examination and clinical history should lead early to the diagnosis of MG with (impending) crisis. The detection of red flags and the dynamic deterioration of symptoms entail admission to the intensive care unit. Due to bulbar symptoms with aspiration and/or respiratory insufficency, early intubation to secure the airway is essential. Therapy includes symptomatic treatment with pyridostigmine or neostigmine and acute causal treatment by immunoadsorption/plasmapheresis or alternatively with immunoglobulins. If used early, intubation may still be prevented and clinical improvement can be achieved within a few days. At the same time, immunosuppression with corticosteroids and azathioprine should be initiated or optimized. For escalation rituximab is an option. The early diagnosis and consequent treatment of infections and other complications such as delirium influence the further course.

## Flow chart SOP myasthenic crisis

**Fig. 1:**
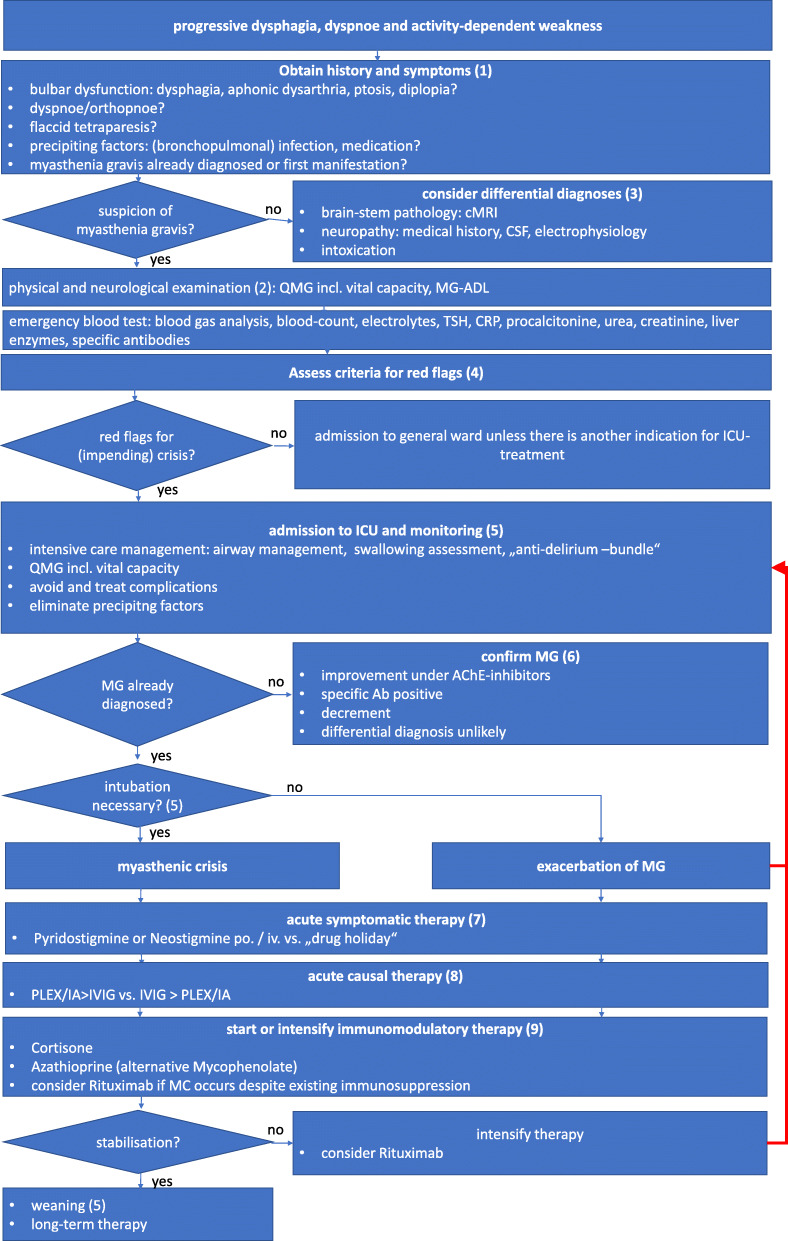
Flow chart - SOP myasthenic crisis

Comments, Explanations, Additions (see footnotes in Figure 1)

1. In most cases, a crisis is preceded by a prodromal syndrome of several days or even weeks with new or aggravated myasthenic symptoms like bulbar and/or generalized, especially respiratory weakness. Typical symptoms to encounter are:
  ptosis increasing in the course of the day
  double vision especially at the end of the day
  difficulties to swallow
  ingestion, cough after eating, and frank aspiration
  leakage (“upward aspiration”) of liquids and food in the nose during the act of swallowing
  fainting and failure of the voice during prolonged speech
  usually weakness of the anterior cervical musculature, with head drop
  breathing with the help of respiratory muscles, orthopnea
  very often pneumonia due to aspiration and signs of sepsis due to decreased ventilation and aspiration

Possible triggers of a crisis or unspecific preceding events are most likely:

- (bronchopulmonary) infections
- treatment with certain medications (see Table 1)
- increase or decrease of cortisone dose / errors in treatment of MG

2. Adding to medical history (trigger?, prodromal symptoms?, MG already known?) clinical evaluation of myasthenic symptoms (in particular bulbar symptoms and / or respiratory insufficiency) is most important. QMG and MG-ADL are most useful scoring systems to measure the severity of deficits and to monitor the clinical course of disease.
3. For any new muscular weakness associated with dysphagia and / or dyspnea or respiratory failure, myasthenia gravis or myasthenic crisis should always be considered in differential diagnosis. Other possible etiologies are listed in Table 2.

**Table 1:** Medications which might worsen myasthenia gravis

Substance group	Example
Steroids, high dose	Dexamethasone, Triamcinolone (also locally)
Gyrase inhibitors	Moxifloxacine
Macrolides	Azithromycine, Clarithromycine, Telithromycine
Lincomycins	Clindamycine
Tetracyclines	Doxycycline
non-Nifedipin-type calcium antagonists	Verapamile
Antipsychotics	Opipramole, Sulpiride

**Table 2:** Extract of possible differential diagnoses of myasthenia gravis and newly occurring and progressive dysphagia respectively

Alternative etiology	Syndrome / diagnosis	Diagnostic
CNS	brainstem-pathology: stroke, rhombencephalitis, multiple sclerosis	• medical history
		• additional symptoms correlating with brainstem-syndrome
		• cMRI
		• cerebral-spinal-fluid (CSF)
intoxication	• cholinergic crisis	• medical history
	o organophosphates	• muscarinergic and nicotinergic symptoms
	o AchE-inhibitors	
		• improvement by atropine
	• Botulism	
		• medical history
	• Botulinum toxin overdose	
		• affection of cranial nerves with tonic pupils
disturbance of the neuromuscular transmission	• Lambert-Eaton-Syndrom	• antibodies (anti-VGKC-Ab)
	• congenital Masthenia gravis	• medical history
		• electrophysiology (increment)
myopathy	• endocrinopathy (hyperparathyreodism, hypo/hyperthyreosis, hyperinsulinism, M. Addison)	• laboratory parameters: TSH, T3/4, CK, potassium etc.
		• specific antibodies
		• medical history
	• hypokaliaemia,	
		• electrophysiology
	• dermato/polymyositis,	
	• toxic/medication (statins, cortisone)	
polyneuropathy / polyradiculopathy	• Guillain-Barré-Syndrome	• CSF
	• Miller-Fisher-Syndrome	• antibodies (anti-gangliosid)
	• intoxication	
		• medical history
	• critical-illness-polyneuropathy	• loss of reflexes and sensory deficits
		• electrophysiology
motoneuron disease	amyotrophic lateral sclerosis	• medical history
		• fasciculations, spastic paresis
		• electrophysiology
		• cMRI

Even though the cholingergic crisis is rare today, due to intoxication with AchE-inhibitors the typical symptoms should still be known: muscarinic overstimulation causes miosis, bradycardia, diarrhea, salivation, warm and red skin and nicotinergic effects are crampi, muscle weakness, fasciculation. However, a differentiation between myasthenic crisis and cholinergic crisis is sometimes difficult (insensitive crisis). Then the probative administration of neostigmine or pyridostigmine as well as atropine under monitoring can help to differentiate the need for AchE-inhibitors or an overdose (also see point 6) [1-10].

4. Red flags for the impending MC are:
  febrile infection in the last 2 weeks treated with antibiotics
  “inverse aspiration”: food and drink get into the nose during swallowing
  insufficient swallowing: coughing or clearing throat after swallowing
  insufficient coughing or coughing impulse
  aphonic dysarthria: typically weakness of phonation during speech with nasal pronunciation (rhinophonia aperta)
  “dropped head”: head falls forward, fixed paresis of the head extensors
  “dropped chin”: lower jaw drops after (longer) chewing
  new facial weakness
  vital capacity < 20 ml/kg body weight, (e.g., < 1500 ml in men or < 1000 ml in women)

## Red flags and dynamic symptom deterioration should lead to admission to an intensive care unit or intermediate care unit

**Table:** 

***CAVE*** *: In addition to aspiration with pneumonia and sepsis, acute life-threatening events occur due to dysphagia with bolus events or ingestion and/or due to respiratory weakness (eg, by coughing) with respiratory insufficiency and consequently hypoxic damage (asphyxia).*

5. The patient with an impending crisis (see “red flags”) should be monitored closely, which means a regular swallowing assessment and regular survey (e.g. every 4-6 h) of the QMG including vital capacity and blood gas analyzes as well as continuous measurement of oxygen saturation regarding respiratory decompensation. For this, admission to an ICU or at least IMC unit with neurologic competence is essential.
  Intensive care management also includes:
  ◦ swallowing assessment / dysphagia therapy (naso-gastric tube), FEES
  ◦ airway-management
    ▪ as long as there is no severe dysphagia and the respiratory situation seems compensated, non-invasive ventilation (NIV) may be considered in order to give the patient the necessary breather and possibly prevent intubation
    ▪ indication for intubation is based on static parameters (see Table 3), but there are no strict cut-off values as they might differ interindivually; more important are deterioration in blood-gas-analysis and dynamics of the decrease in vital capacity as well as severe dysphagia or (silent) aspiration

**Table 3:** Parameters for bedside ventilation during myasthenic crisis [6]

Criterion/indication	Normal	Intubation	Weaning	Extubation
Vital capacity (ml/kg body weight)	> 60	< 20	> 15	> 25
Negative airway pressure (cm H_2_O)	> 70	< 30	> 20	> 40
Positive airway pressure (cm H_2_O)	> 100	< 40	> 40	> 50
    ▪ choose pressure-regulated ventilation
    ▪ weaning: episodes of spontaneous breathing with positive airway pressure (CPAP) in extended intervals in a structured manner
    ▪ extubation: stable and satisfactory ventilation-related parameters (see Table 3), sufficient cough and adequate swallow at least for thickened liquid or mushy food
    ▪ consider early elective tracheotomy in prolonged crisis
  ◦ eliminate and treat precipiting factors and complications like infection or electrolyte disturbances, consider the impact of comorbidities
  ◦ delirium
    ▪ day-structuring activities, establish day-night-rhythm, physio- and ergotherapy (“anti-delirium bundle”)
    ▪ lorazepam for anxious-agitated patients
    ▪ if necessary: phenothiazine, haldol or low-potency neuroleptics
6. Since not all diagnostic steps can be taken during the acute situation, a further approach should be taken during parallel intensive care treatment to confirm the diagnosis of MG (crisis as first manifestation of the disease), to detect possible precipiting factors of the crisis, and to rule out differential diagnoses (see Table 2):
  electrophysiological examination:
    ◦ repetitive stimulation to detect decremental response
    ◦ exclusion of neuropathy (e.g., Gulliain-Barre-Syndrome, Miller-Fisher-Syndrome, critical-illness-neuropathy)
    ◦ exclusion of myopathy (e.g., rhabdomyolysis)
  laboratory:
    ◦ blood-cell-count, electrolytes including potassium, TSH, CRP, procalcitonine, urea, creatinine, liver enzymes
    ◦ blood culture
    ◦ specific antibodies (anti-AChR-ab, anti-Musk-ab etc.)
  imaging:
    ◦ chest X-ray: pneumonia?
    ◦ consider CT or MRI of chest regarding thymus pathology
    ◦ consider cMRI with Gd to rule out brainstem pathology
  pharmacologic testing:
    ◦ *ex juvantibus* AChE-inhibitors (former “tensilone test”): for example with neostigmine or pyridostigmine iv. (keep atropine ready as antidote!)
7. Therapy of MC and imminent MC is multidimensional including symptomatic acute treatment, causal acute treatment, initiation or modification of long-term immunosuppressive therapy and specialized intensive care management.
  Symptomatic acute treatment:
  ◦ pyridostigmine (Mestinon®) p.o. 3-6x60mg, max. 540 mg/d **or**
  ◦ pyridostigmine iv. (equivalent: orally:parenterally ~ 30:1)
    ▪ 360 mg/d p.o. equals to 12 mg/d i.v., max. 24 mg/d
    ▪ bolus 1–3 mg followed by 0.5–1 mg/h
  ◦ alternatively:
    ▪ neostigmine iv. (equivalent:: orally:parenterally = ~ 80:1)
    ▪ 360 mg/d Pyridostigmine p.o. = 4.5 mg/d neostigmine i.v.
    ▪ starting dose 6–12 mg/24 h, adjust 0.2–0.8 mg/h, bolus of 0.5 mg possible
  ◦ atropine 0.5–1 mg s. c. oder iv. against side effects (bronchial secretion)
  ◦ consider “drug-holiday” when intubated and not breathing spontaneously
8. Effects of causal acute therapy can be observed after a few days usually (see Table 4). Indication for each regime depends on whether there is a crisis or an exacerbation and on complications.
  ◦ plasma exchange (PLEX) or immunoadsorption (IA)
    ▪ first-line therapy of crisis

**Table 4:** Latency of myasthenia therapies

Therapy	Latency
PLEX/IA	few days
IVIG	few days (“Dip” possible?)
Cortisone	3–4 weeks (“Dip” frequent!)
Azathioprine	6–12 months
Mycophenolate	6–12 months (???)
Rituximab	2–3 months
Thymektomy	months-years
    ▪ 5–6 or even more treatments
    ▪ effect after a few days
  ◦ polyvalent immunglobulines (IVIG)
    ▪ first choice for exacerbation / imminent crisis
    ▪ 0.4 g/kg body weight per day for 5 consecutive days or 1,5-2 g/kg body weight (bw) for 2(− 3) days
    ▪ effect after a few days
9. Long term immunosuppressive therapy should be started also during crisis/exacerbation already although effects appear after several weeks (cortisone) or months. It contributes to long-term stabilization.
  ◦ cortisone
    ▪ effect after 1 month
    ▪ CAVE: “dip” with initial deterioration possible, the more critical the clinical situation the lower should be the starting dose!
      exacerbation:
        ◦ increasing dosage, for example Prednisolone 10–20–40–60 mg/d
        ◦ increase weekly in order to avoid initial deterioration (“dip”)
        ◦ decrease in similar steps but every 2–3 week intervals to 20 mg/d, then choose longer intervals and smaller steps depending on clinical condition
      crisis (artifical ventilation):
        ◦ 100 mg Prednisolone
        ◦ reduce every 10 days by 10-20 mg to 30 mg/d, thereafter at longer intervals and with decreasing dose steps
  ◦ azathioprine, started parallely with cortisone:
    ▪ start when septic conditions are ruled out
    ▪ 1st week 50 mg/d – 2nd week 100 mg/d – 3rd week 150 mg/d or 2,5 mg/kg bw
    ▪ further dosage depends on laboratory parameters: 6–8 weeks after initiation absolute lymphocyte count should be 0,6–1,0/nl while whole leucocyte count should be > 3,0/nl and transaminases less than 5-fold of upper limit
    ▪ alternative (e.g. TPMT-deficiency): mycophenolate (MMF)
    ▪ in case of myasthenic crisis despite existing (and effectively ingested) immunosuppressive therapy: escalation with rituximab for both AChR-Ak positive and MuSK-Ak positive myasthenia
      several dosage schemes; e.g. 1 g rituximab given twice at 14-day intervals
      repeat after 1 year or after the CD19 positive cells have risen to the measurable range or after recurrence of symptoms

## Abbreviations

ab: Antibody
AChE-inhibitor: Acetylcholin-esterase inhibitor
AChR: Acetylcholin receptor
cMRI: Cranial magnet resonance tomography
CSF: Cerebrospinal fluid
FEES: Fiberoptic endoscopic evaluation of swallowing
IA: Immunoadsorption
IVIG: Immunglobulines
MC: Myasthenic crisis
MG: Myasthenia gravis
MMF: Mycophenolate mofetil
PLEX: Plasma exchange
